# Supplementary material for: Thalamic nucleus reuniens coordinates prefrontal-hippocampal synchrony to suppress extinguished fear
Source: Nat Commun. 2023 Oct 17;14:6565. doi: 10.1038/s41467-023-42315-1 (PMC10582091; doi:10.1038/s41467-023-42315-1)
Supplement: Supplementary file 1 — Supplementary Information [file 41467_2023_42315_MOESM1_ESM.pdf]

Supplemental Information for:

Thalamic nucleus reuniens coordinates prefrontal-hippocampal synchrony to suppress extinguished fear

Michael S. Totty[1,2], Tuğçe Tuna[1,2], Karthik R. Ramanathan[1,2], Jingji Jin[1,2], Shaun E. Peters[1], and Stephen Maren[1,2*]

[1] Department of Psychological and Brain Sciences, Texas A&M University, College Station, Texas

[2] Institute for Neuroscience, Texas A&M University, College Station, Texas

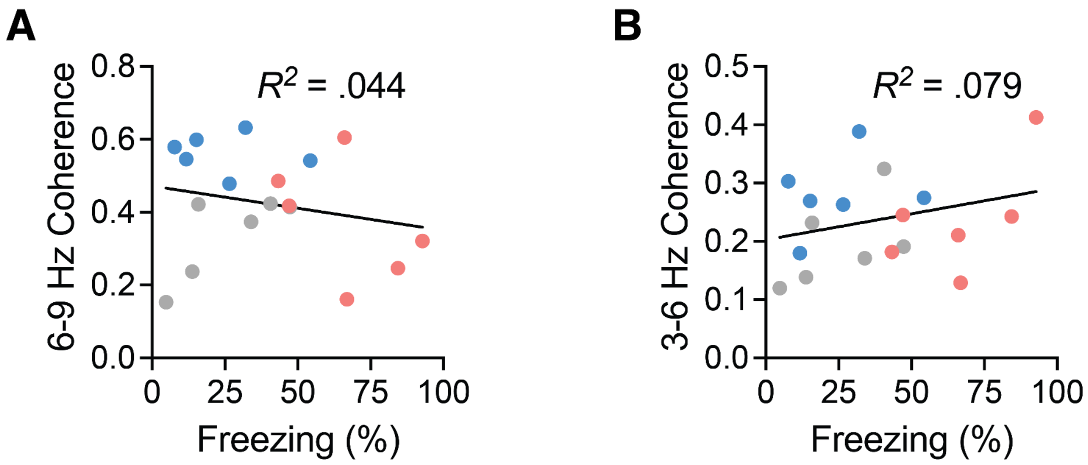

Supplemental Figure 1: PFC-HPC coherence cannot be explained by differences in freezing behavior. (A) Linear regression analysis showing that peak coherence in both the 6-9 Hz (A; non-significant slope: $F_{1, 16} = 0.045$, $p = .152$) and 3-6 Hz (B; non-significant slope: $F_{1, 16} = 0.079$, $p = .082$) frequency range has a weak relationship with the percentage of time animals exhibited freezing behavior across experimental timepoints. Source data are provided as a Source Data file.

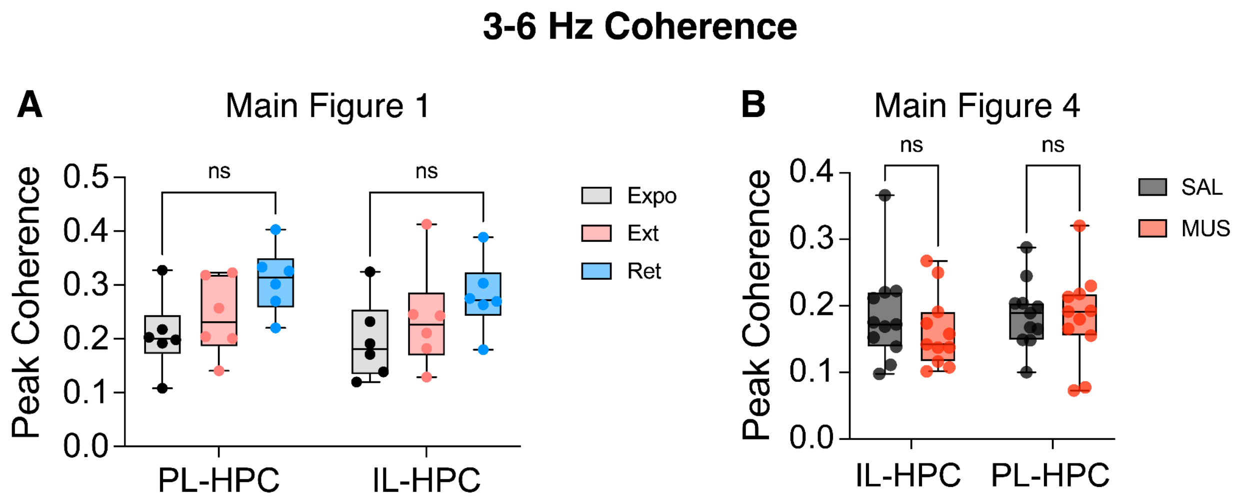

Supplemental Figure 2: PFC-HPC coherence in the 3-6 Hz frequency range. (A) Related to Figure 1, although 3-6 Hz peak coherence does appear to increase across test sessions (main effect of Day: $F_{2, 20} = 4.88$, $p = .019$), Tukey's post hoc comparisons did not reveal any significant effects (all $p > .06$). (B) Related to Figure 4, 3-6 Hz coherence was not affected by muscimol inactivation of the RE (main effect of Drug: $F_{1, 20} = 0.305$, $p = .587$). Boxplots represent mean plus minima and maxima with lower and upper quantiles. Source data are provided as a Source Data file.

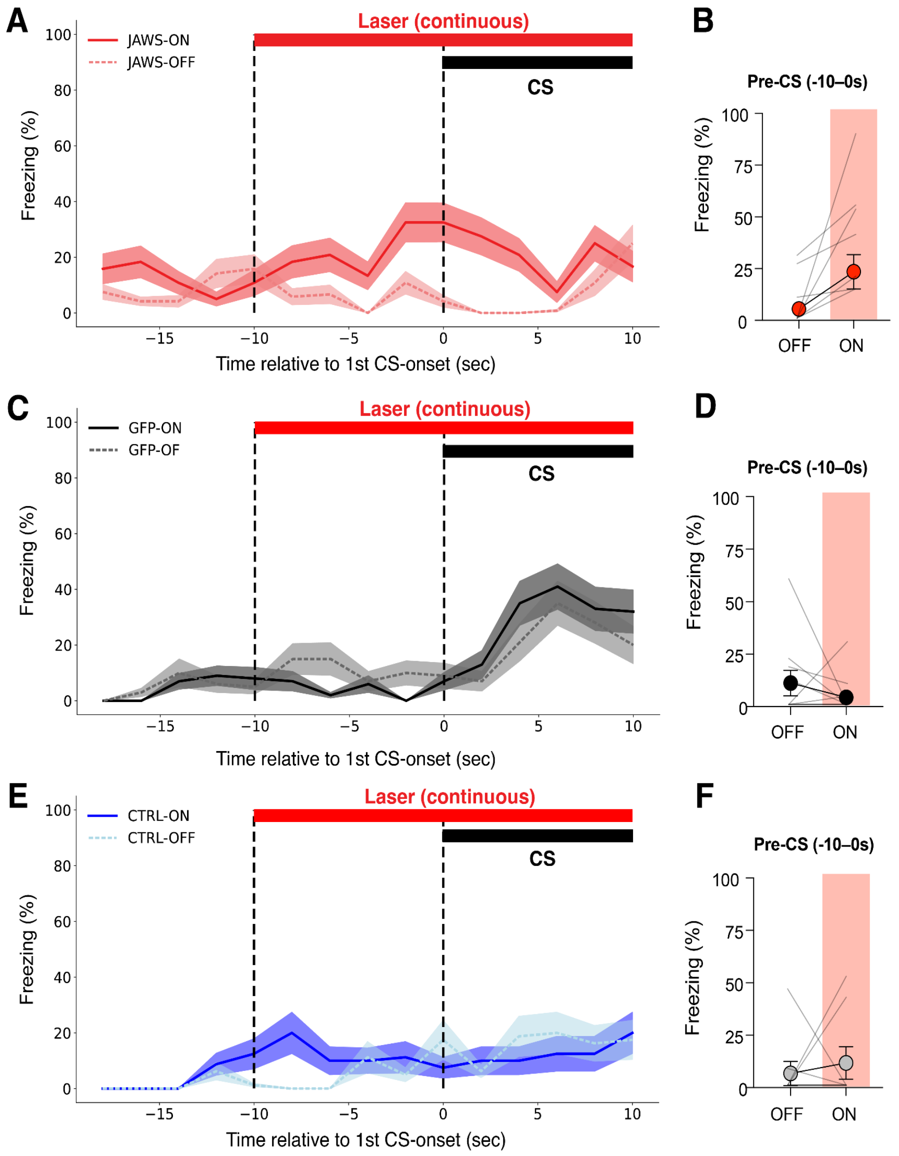

Supplemental Figure 3: The evolution of freezing behavior during the first CS trial of Retrieval testing shown in Figure 4. (A,C,E) Two-second bins of freezing behavior were averaged starting from 20 seconds preceding the first CS onset to the 10 second period after CS onset for Jaws, GFP, and control (CTRL) groups during both laser ON and OFF conditions. Red laser illumination began 10 seconds prior to CS presentation. To determine if the laser had any effect before CS trials began, the freezing during this 10 second pre-CS period was averaged for each group. Although there was no significant Laser x Group interaction ($F_{2, 27} = 2.379$, $p = .112$), planned comparisons using Fisher's LSD show that red laser stimulation increased freezing in the Jaws group prior to CS presentations ($p = .027$), but not the GFP ($p = .426$) or CTRL groups ($p = .599$). All data are means ± s.e.m.s; $*p < 0.05$. All plots represent mean ± s.e.m.s; $*p < 0.05$. Source data are provided as a Source Data file.

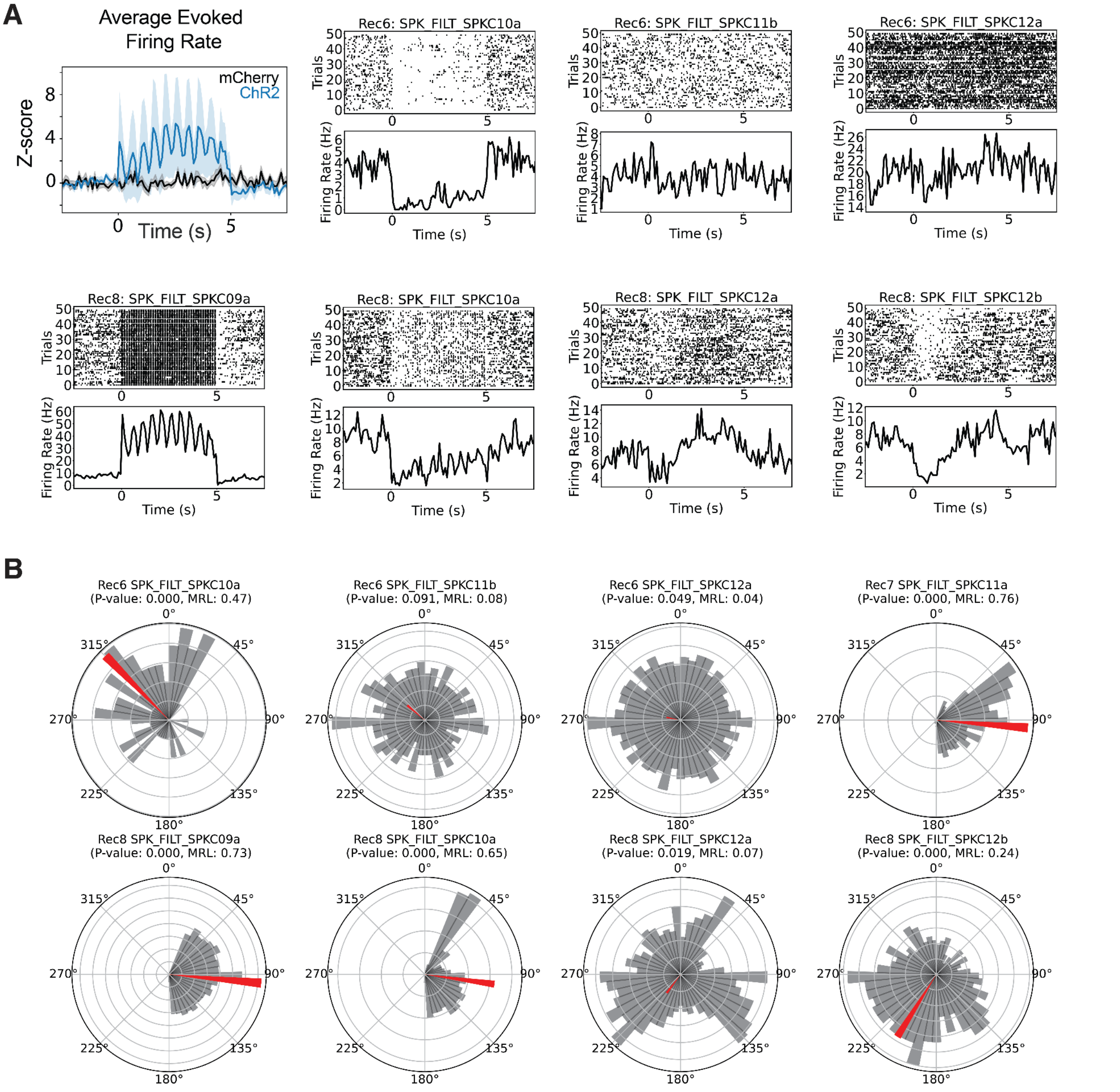

Supplemental Figure 4: Electrophysiological data related to Figure 6. (A) Average light-evoked firing rate in ChR2 and mCherry rats across all 8 neurons. Raw raster plots and average light-evoked firing over trials for neurons recorded from ChR2-expressing rats shows mixed responses with some neurons showing strong excitatory and others showing an initial suppression before becoming entrained to the phase laser stimulation. (B) Rose plots showing the distribution of spikes in individual ChR2 units in relation to the phase of blue-laser stimulation. The strength of entrainment is determined by the mean resultant length (i.e., length of the red bar), a measure of concentration in circular distributions. Sine wave stimulation reached max intensity at 90°. Source data are provided as a Source Data file.

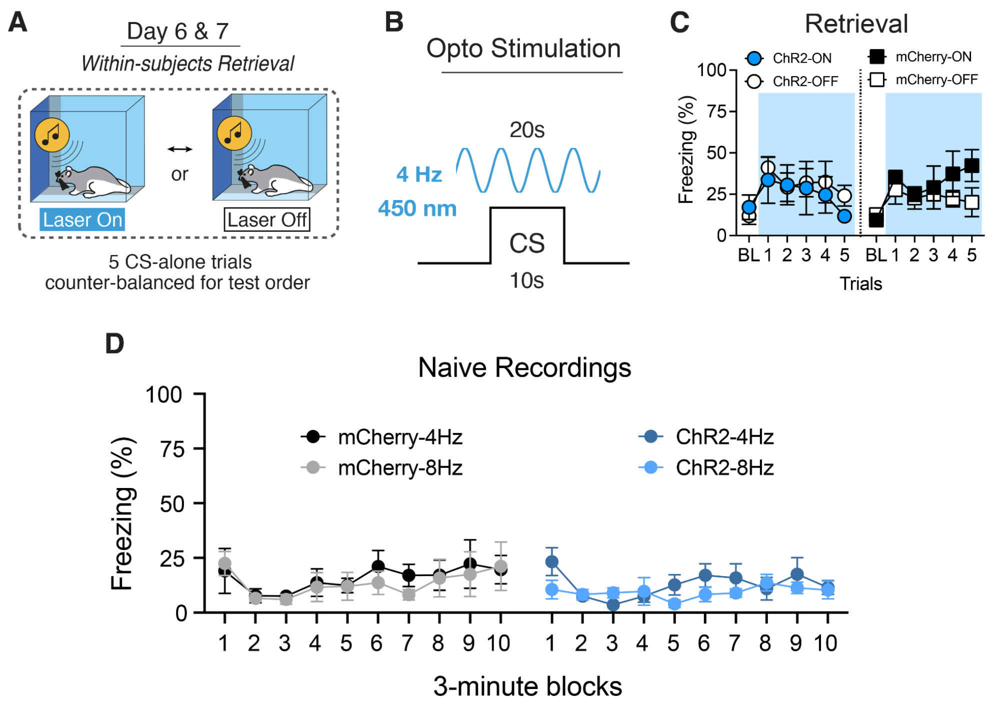

Supplemental Figure 5: Behavioral data related to Figure 6. (A) After the behavioral testing outlined in Figure 6, rats received an additional test to determine if 4-Hz sinusoidal stimulation of the RE impaired extinction retrieval. (B) Similar to the 8-Hz stimulation protocol, 4-Hz laser stimulation began 5 seconds before CS presentations and was turned off 5 seconds after CS termination. (C) Freezing data showing that 4 Hz stimulation had no effect on freezing behavior (no main effect of Group: $F_{1, 8} = 0.007$, $p = .936$; no main effect of Laser: $F_{1, 8} = 0.345$, $p = .573$; no Group x Frequency interaction: $F_{1, 8} = 3.24$, $p = .10$) in either the ChR2 (n = 5, 3 male and 2 female) or mCherry-expressing rats (n = 5; 2 male and 2 female). (D) Importantly, neither 4 nor 8 Hz stimulation had any effects on freezing behavior during naive recordings prior to behavioral testing (no main effect of Group: $F_{1, 90} = 3.24$, $p = .075$; no main effect of Frequency: $F_{1, 29} = 3.51$, $p = .064$; no Group x Frequency interaction: $F_{1, 90} = 0.12$, $p = .733$), suggesting that all effects shown in Figure 6 are not simple due to changes in performance. All data are means ± s.e.m.s. Line plots represent mean ± s.e.m.s. Source data are provided as a Source Data file.

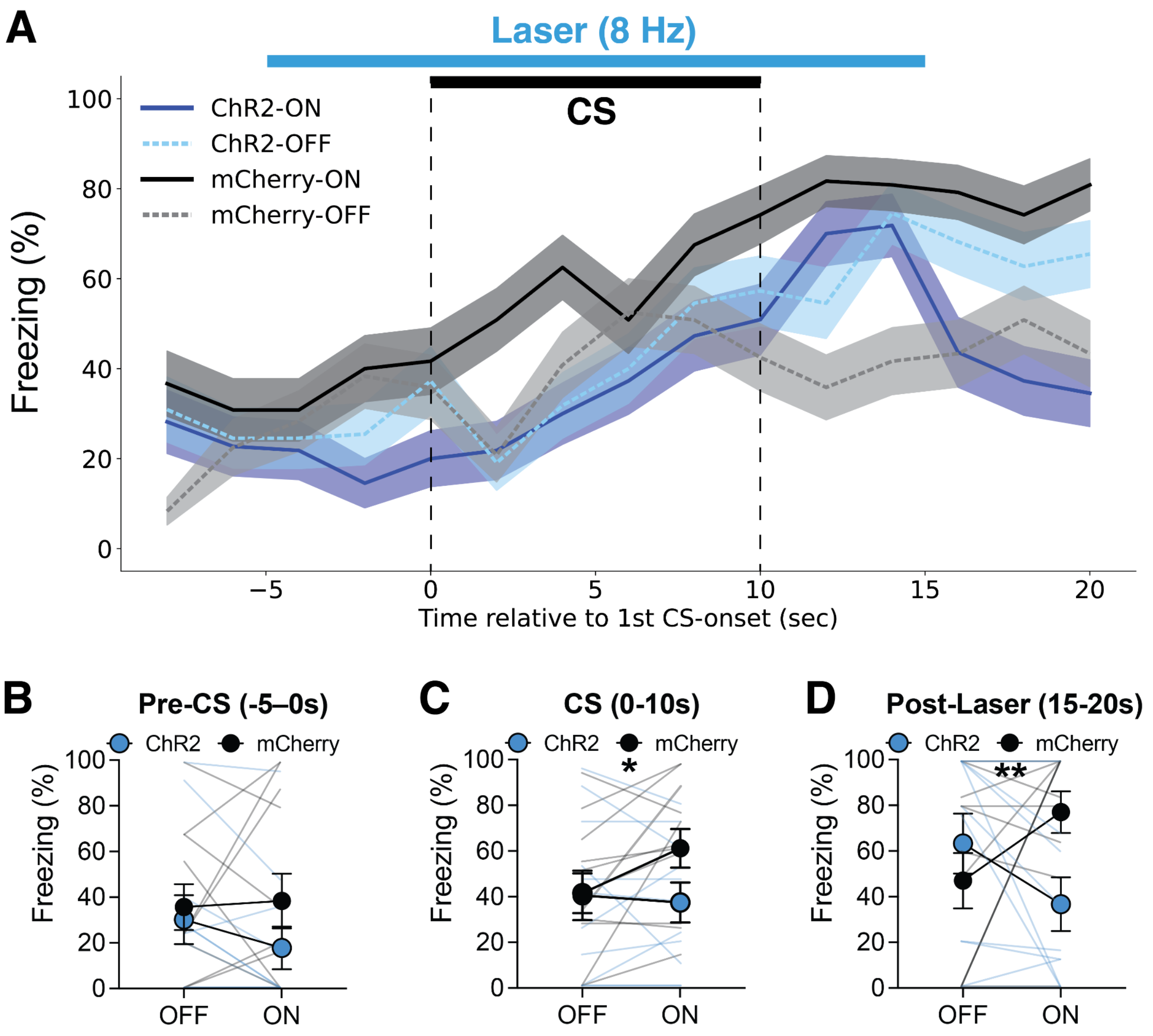

Supplemental Figure 6: The evolution of freezing behavior during the first CS trial of Renewal testing shown in Figure 6. (A) Time course of freezing behavior in ChR2 and mCherry-expressing rats during both laser ON and OFF test sessions in relation to Laser and CS duration. (B) Laser presentation did not affect freezing behavior during the 5 seconds prior to the CS (Pre-CS period; Laser x Group interaction: $F_{1,\,20} = 2.25$, $p = .149$). During the CS (C), mCherry rats show increased freezing when the Laser was turned ON (CS period; Laser x Group interaction: $F_{1,\,20} = 4.82$, $p = .040$). Comparatively, (D) ChR2-expressing rats showed a quick reduction in fear following CS and laser termination (Post-CS period; Laser x Group interaction: $F_{1,\,20} = 9.35$, $p = .006$). All data are means ± s.e.m.s; $*p < 0.05$; $**p < 0.01$. Line plots represent mean ± s.e.m.s. $*p < 0.05$; $**p < 0.01$. Source data are provided as a Source Data file.

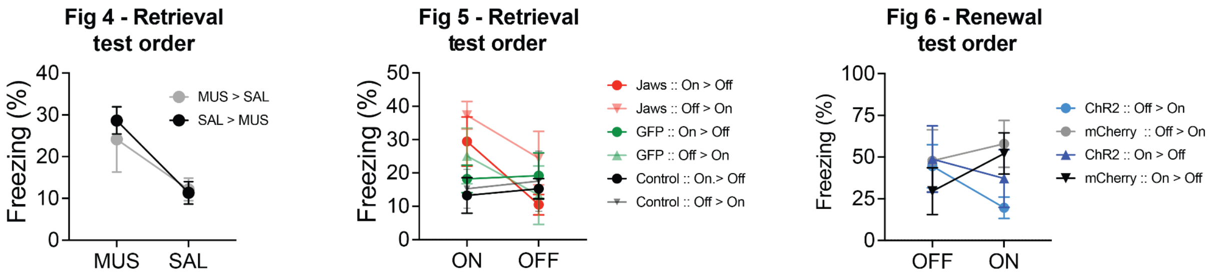

Supplemental Figure 7: Within-subjects behavioral data from Figures 4, 5, and 6 plotted by test order. There were no test-order effects in any of the within-subjects behavioral tests in either Figure 4 (main effect of Test Order: $F_{1, 9} = 0.171$, $p = .688$), Figure 5 (main effect of Test Order: $F_{5, 25} = 1.208$, $p = .3342$), or Figure 6 (main effect of Test Order: $F_{1, 18} = 0.002$, $p = .968$). This is visually shown by the similar line slopes in animals of the same group regardless of test order (e.g., On > Off or Off > On). All data are means ± s.e.m.s. Line plots represent mean ± s.e.m.s. Source data are provided as a Source Data file.
